# The Stereoscope

**Published:** 1898-12-17

**Authors:** 


### THE STEREOSCOPE.

Dr. Mackenzie Davidson has done well in drawing attention to that far too much neglected instrument, the stereoscope. Everyone knows the difficulty that there often is in properly interpreting ordinary photographic illustrations of pathological appearances. Much, however, that appears meaningless where everything is depicted in the same plane becomes at once instructive as soon as it stands out in the stereoscope, and occupies its true position in regard to its surroundings. The admirable stereoscopic photograph of a case of small-pox which accompanies his paper[1] serves well to show how much more illustrative the combined pictures are than either of them separately, the pustules standing out plump and fat upon the patient's face. Dr. Davidson specially insists on the utility of the stereoscopic method in skiagraphy, and there can be no doubt that he is right. Not only may stereoscopic skiagraphs be so arranged as to do away with the apparent distortion which is so often seen in X-ray photographs, but they give most useful information as to the depth of, say, a foreign body, which can only otherwise be obtained by careful comparison of two plates.

[1] Brit. Med. Jour., Dec. 3.

